# Impact of Different Glidepath Techniques on the Overall Performance of WaveOne Gold in an Artificial S-Shape Canal

**DOI:** 10.3390/dj12060182

**Published:** 2024-06-13

**Authors:** Vlad Mircea Lup, Olivia Andreea Marcu, Carlo Gaeta, Gabriela Ciavoi

**Affiliations:** 1 Doctoral School of Biomedical Sciences, University of Oradea, 410087 Oradea, Romania; 2 Department of Preclinical Disciplines, Faculty of Medicine and Pharmacy, University of Oradea, 410087 Oradea, Romania; 3 Unit of Periodontology, Endodontic and Restorative Dentistry, Department of Medical Biotechnologies, University of Siena, 53100 Siena, Italy; odontoiatriagaeta@libero.it; 4 Faculty of Medicine and Pharmacy, University of Oradea, 410087 Oradea, Romania; gciavoi@uoradea.ro

**Keywords:** glide path preparation time, ProGlider, root canal preparation, shaping time, WaveOne Gold, WaveOne Gold Glider

## Abstract

Objectives: The article’s aim is to test if rotary or reciprocating glide path influences the overall performance of WaveOne Gold in S-shaped canals. Methods: Sixty endo training blocks with an S-shape curvature were divided into three groups based on the glide path method used: no glide path; glide path preparation with ProGlider; glide path preparation with WaveOne Gold Glider. All blocks were then shaped with WaveOne Gold Primary. The time for shaping, the incidence in reaching working length and the number of pecking motions were recorded. ANOVA with Turkey’s test was used, and the *p*-value was set to 0.05. Results: WaveOne Gold Primary reached working length faster in the control group when comparing total working times. No significant differences in the ability of the WaveOne Gold Primary to reach working length in all groups (*p* > 0.05). The mean number of pecking motions was higher in the control group compared to other groups. Conclusions: No significant differences in the time needed to achieve a glide path between Proglider and WaveOne Gold Glider. WaveOne Gold Primary can shape a double curved canal faster if a glide path is present but takes less time to reach length if it is the only file used. No difference in the ability to reach working length.

## 1. Introduction

In the last decades, the use of nickel-titanium (Ni-Ti) rotary instruments has become a must for root canal preparation because it is a safe, efficient, and a fast way to achieve a desirable shape inside the root canal [1]. However, one of the biggest problems with these files is the risk of separation inside the root canal due to flexural and torsional stress [2]. Studies have shown that higher torsional stress is associated with a wider area of contact between the dentinal walls and the body of the instrument, as well as when the canal diameter is smaller than the non-cutting tip of the instrument, better known as taper-lock [3]. The creation of a glide path before using Ni-Ti instruments has been shown to reduce this stress [4], decreasing the rate of instrument fractures, and also avoid several procedural errors such as canal transportation and ledge formation [5].

For a long time, glide path was created manually, with stainless steel K-files, as it was a reliable technique [6], and clinicians were more in control by “feeling” the file. Some authors [7] considered a glidepath was present when a size 0.10 K-file moved freely inside the canal, whereas others [8] stated that a safe glide path is one where a size 0.15 K-file slides freely to length without any need for rotation. But establishing a glide path with hand files can be time-consuming [2], technique-sensitive [9], and prone to poor outcomes [10]. A safer and less invasive method for creating a glide path has been reported to be the use of a small stainless steel K file for initial canal scouting and negotiation, followed by a more flexible, stronger, and efficient Ni-Ti rotary glide path file [11]. Thus, a glide path allows clinicians to understand and respect the original canal anatomy, prepares the canal to receive rotary files, and makes root canal shaping safer and more effective [12].

Since then, quite a big number of glide path specific Ni-Ti instruments have been introduced on the market, each with their unique cross-section design, taper, tip size, alloy treatment and kinematics, to make glide path safer, quicker, and reliable [13]. With the introduction of M-Wire, flexibility has been improved and cyclic fatigue resistance has been increased by up to 400% [14].

One of these specific glide path instruments is the ProGlider (PG), a single use rotary glide path instrument, with a diameter of 0.16 at its tip, and increasing tapers from 2% to 8% along the active part of the instrument.

Considering the recent studies in reciprocating movement state that reciprocal files have a greater fatigue resistance, a higher cutting efficiency and produce a minimum damage to dentinal walls [15], a single reciprocating file for glide path management was developed, the WaveOne Gold Glider (WOGG). The Gold Glider has a semi-active tip of 0.15 in diameter, and a parallelogram cross section with progressively increasing tapers from 2% to 6%.

The kinematics of both rotary and reciprocating motions have been intensively studied, to determine the cyclic and torsional fatigue resistance of various Ni-Ti instruments [16].

Reciprocating motion is also highly appraised in shaping Ni-Ti files, as manufacturers claim an increase in resistance to separation. One of these shaping systems is the WaveOne Gold (WOG), that has been proven to have an increased resistance to separation by several studies [17].

Although glide path is still an important step in canal shaping with rotary files, the impact of glide path preparation on reciprocating systems remains unclear [18]. On one hand, studies have shown that it is possible to shape a straight or moderately curved root canal with reciprocating instruments up to the full working length without a glide path preparation [19,20,21], but on the other hand, another article [22] examined the influence of glide path preparation on the failure rate of WaveOne instruments, and found out that the total canal preparation time, and number of simulated canals prepared with WaveOne instruments was influenced by the method of glidepath preparation.

The purpose of this study was to compare the preparation time of a glide path with continuous rotation and reciprocation movements, and to see if glide path had an influence on the shaping time and ability to reach full working length of WaveOne Gold Primary File in simulated S-shaped canals.

## 2. Materials and Methods

A power calculation was performed with G*Power 3.1 (Heinrich Heine University, Dusseldorf, Germany) software. For a one-way analysis of variance, the software indicated that the total sample size to yield a statistical power of at least 0.8 with an alpha of 0.05 and a medium effect size (d = 0.5) is 42 (14 samples in each group).

For this study, a number of 60 (*n* = 60) plastic blocks (Dentsply Sirona, Ballaigues, Switzerland) with a simulated S-shape canal were used. The standard artificial canals were 16 mm in length, had a 2% taper and a double, S-shape curvature. The first curve had a radius of 3 mm, a 30° angle of curvature, and was situated at 8 mm from its terminus, whereas the second one had a radius of 2 mm, a 25° angle of curvature and was located at 2 mm from the terminus.

The blocks were then divided into 3 groups (*n* = 20 each).

The control group, no glide path prepared (NGP), 20 blocks—the operator scouted the canals with a size 0.10 stainless steel K-file (VDW, Munich, Germany) up to the working length. Irrigation was performed with saline solution to remove any debris accumulated.

The ProGlider Group (PGG), 20 blocks—all canals in this group were scouted by the same operator with a new size 0.10 stainless steel K-file up to the working length. After that, a glide path was created with new PG files (Dentsply Sirona, Ballaigues, Switzerland), one per block, according to the manufacturer protocol in the corresponding program of the endodontic motor that was used, the X-Smart Pro + (Dentsply Sirona, Ballaigues, Switzerland). Irrigation was performed with saline solution to remove any debris accumulated, with the protocol of Irrigation, Recapitulation, Irrigation (IRI).

The WaveOne Gold Glider Group (WOGGG), 20 blocks—all canals in this group were scouted by the same operator with a new size 0.10 stainless steel K-file up to the working length. After that, a glide path was created with new WOGG files (Dentsply Sirona, Ballaigues, Switzerland), one per block, according to the manufacturer protocol in the corresponding program of the endodontic motor that was used, the X-Smart Pro +. Irrigation was performed with saline solution to remove any debris accumulated, with the IRI protocol.

After the blocks were prepared according to each group’s specifications, all 60 blocks were shaped by a single operator using new WOG Primary files (one per block) according to the manufacturer protocol in the corresponding program of the endodontic motor that was used, the X-Smart Pro +. Irrigation was performed with saline solution to remove any debris accumulated, with the IRI protocol.

For the PG group the protocol used was as follows:

- The simulated canal was explored with a new #10 K-file;
- Working length was established;
- The endodontic motor was set on PG program, using 300 RPM and a torque of 4 Ncm;
- In the presence of saline solution, the PG file was used to passively follow the canal, in one or more passes until working length was reached;
- Working length was reconfirmed.

For the WOGG group the protocol used was as follows:

- The simulated canal was explored with a new #10 K-file;
- Working length was established;
- The endodontic motor was set on the WOGG program that used the manufacturer’s settings for speed, torque, and angles of reciprocation;
- In the presence of saline solution, the WOGG file was used with gentle inward pressure so the file would passively follow the secured canal in one or more passes until working length was reached;
- Working length was reconfirmed.

For the shaping of all simulated canals the WOG Primary file was used as follows:

- The endodontic motor was set on WOG Primary program that used the manufacturer’s settings for speed, torque, and angles of reciprocation;
- In the presence of saline solution, the file was used with gentle inward pressure and was let to passively progress inside the canal with in-and-out pecking motion;
- After progressing 2–3 mm (about three pecks) of the canal the file was removed and cleaned, and the canal was irrigated following the IRI protocol;
- The pecking motions were repeated until the instrument reached full working length, separated, blocked the canal, or created a iatrogenic error (unpassable ledge, via falsa).

To avoid intra-operator variability, all procedures described were performed by a single experienced operator.

The glide path preparation time, in seconds, for each block in the PG and WOGG groups was recorded with a Seiko high precision chronometer (Seiko Holding Corporation, Japan). The time for irrigation protocols was not recorded, so the chronometer was started every time the file went inside the canal, and was paused, the moment the file left the canal.

The shaping preparation time, in seconds, for each block was recorded with a high precision chronometer. The time for irrigation protocols was not recorded, so the chronometer was started every time the file went inside the canal, and was paused, the moment the file left the canal.

To prevent discrepancies in measuring the time in seconds, an assistant was trained to start and stop the chronometer when needed.

The failure of the WOG Primary file to reach full working length, the preparation errors such as ledge formation, the mean number of pecking motions and the incidence of instrument separation were also recorded and analyzed.

Statistical analysis was performed on the statistical package SPSS24 (Armonk, NY, USA: IBM). A Shapiro-Wilk test is performed to asses normality of data distribution and ANOVA with Turkey’s test was used in order to compare the level of significance among all pairs of the three groups for parametric data, and the Student’s t-test was applied when differences were tested only among two groups. A significance level of 0.05 was considered in all the analysis.

**Figure 1:**
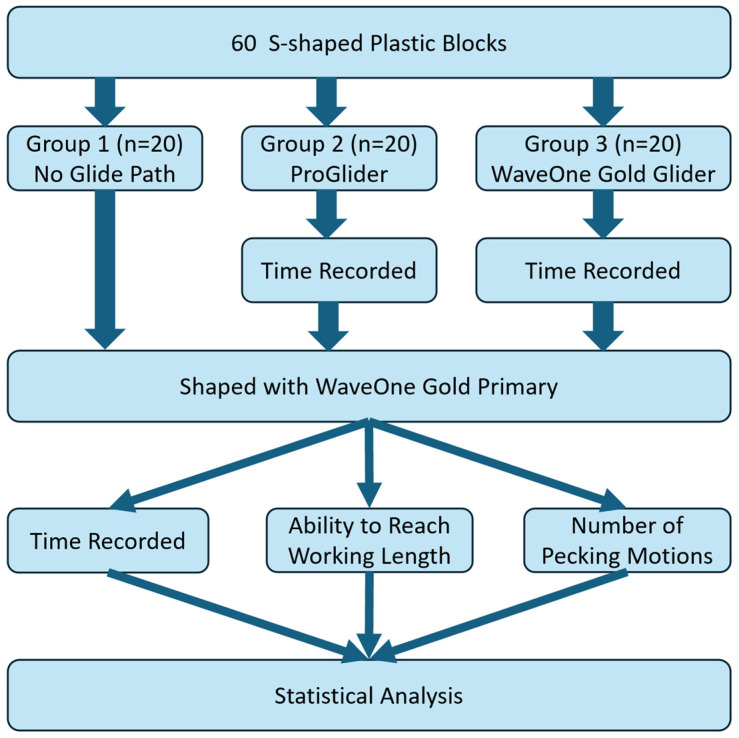
Methodology flowchart.

The workflow methodology is presented in Figure 1.

## 3. Results

The descriptive statistics of the glide path preparation time (GP_time) are presented in Table 1. There are no significant differences in the mean GP_time between the PG and WOGG groups with an average of 5.20 (±1.38) seconds compared to 4.55 (±0.88).

The descriptive statistics for shaping time of the WOG Primary instrument (Shape_time), the total time needed for shaping (Total_time), the incidence in reaching full working length (RFWL), and the number of pecking motions (NPM) needed to reach full working length, are depicted in Table 2. The statistical tests confirmed significant differences among mean value of all three groups under investigation when looking at Shape_time, Total_time, and NPM. There are no significant differences in RFWL as the highest incidence rate is reached in the PG and WOGG groups with a rate of 0.95 while the smallest rate is reached in the NGP group with a rate of 0.85.

Variables follow a normal distribution (GP_time *p*-value = 0.4, Total_time *p*-value = 0.08, RFWL *p*-value = 0.1, NPM *p*-value = 0.32).

The results of Turkey’s test for multiple comparison can be consulted in Table 3. Shape_Time is significantly greater in the NGP group compared to WOGG. Total_Time is significantly greater in the PG group compared to NGP group.

In NPM there were significant differences when NGP and PG or NGP and WOGG groups are compared.

## 4. Discussion

Endodontic training blocks are often used on in vitro studies to evaluate different parameters of endodontic instruments because it is easier to standardize the experimental conditions [23]. In the present study we used these blocks to determine if a glide path preparation had an influence on the shaping parameters of WOG Primary instrument.

The parameters analyzed in this study were the time in seconds needed to achieve a glide path with a continuous rotation single file (PG) and a reciprocating single file (WOGG), the time in seconds needed for the WOG Primary file to reach working length with or without a prior glide path and its ability to do so depending on glidepath, and the number of pecking motions needed to reach working length.

When comparing the glide path preparation times we found out that the mean preparation time for the WOGG is slightly shorter than for the PG, but not statistically significant. The reason may be that WOGG has a heat treatment applied to it which increases its resistance to failure due to cyclic fatigue when compared to M-wire files [24,25]. As a result, WOGG is more flexible than the PG and can negotiate double curvatures with more ease. The authors are unaware of any other study comparing the preparation times of these two files in order to compare results. Previous studies showed that rotary glide path files need less time than manual K-files [22,26,27,28], and single file systems for glide path preparation are faster than multiple instrument file systems [29,30,31].

Analyzing the shaping time of the WOG Primary returned no surprising results. The time needed to shape the double curved canal is significantly longer when no glide path is present because single file glide path systems are manufactured with a progressive taper, and so they previously enlarge the coronal and middle third of the canal. That way, the subsequent shaping file needs less time to progress. Similar results could be observed in other studies as well [22,32,33]. Although the mean shape time in the WOGG group is shorter than in PG group the results are not statistically different. Another article [32] also found no statistically significant differences when comparing same parameters even though their shape time was lower in the PG group compared to the WOGG group.

When comparing the total time needed to shape a canal, we found out that the mean total time for the NGP group was significantly lower compared to PG group, and lower compared to WOGG group. Even though it takes longer for WOG Primary to reach working length if no glide path is present, the time needed to perform a glide path raises the total time needed for shaping.

The reason time discrepancies occurred in the same group (min time vs. max time) is that the complex anatomy of the blocks had an impact on the integrity of the shaping files. That means that some files exhibited deformations during the shaping process and thus, deformed files needed more time to completely shape the canal.

**Figure 2:**
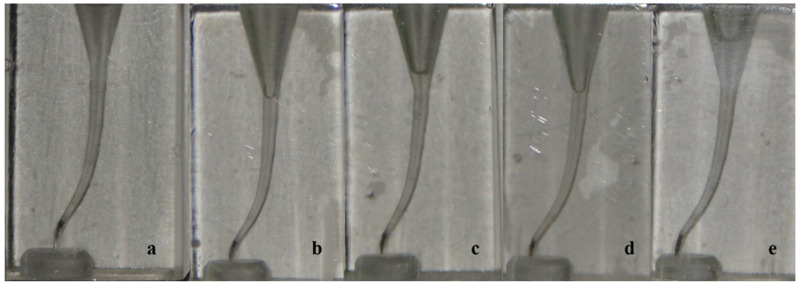
Samples with separated instruments—(**a**) PG 16; (**b**) WOGG 19; (**c**) NGP 4; (**d**) NGP18; (**e**) NGP 20.

In terms of the ability to reach working length we found no significant statistical differences among groups. In total there were five instrument separations, as follows: both PG and WOGG groups had one situation where WOG Primary fractured (PG 16 and WOGG 19) while NGP group had three situations where WOG Primary fractured (NGP 4, 18, and 20) (Figure 2). All fractures occurred in the apical curvature. Even though the WOG Primary is working and removing debris along the whole length of the instrument, the main work load is situated in the apical portion with the greater taper, WOG Primary being a file with decreasing tapers. Therefore the file is subjected to more loading on its apical third especially when no prior glide path is present. That is why a greater failure rate is expected in the NGP group.

The NPM is significantly higher in the NGP group compared to PG and WOGG groups. When Berutti et al. [33] studied root canal anatomy preservation using WaveOne files, they also showed that a previous glide path reduces the NPM to reach working length by WaveOne instruments. That means that the file is working less and is subjected to less load, reducing the incidence of separation. However, giving that the WOG files are meant as a single use instruments, no matter the load they are subjected to, they should be discarded after use.

## 5. Conclusions

Within the limitations of this study, it can be concluded that even though the WaveOne Gold Glider can achieve a faster glide path than the ProGlider, the results are not statisticly significant. WaveOne Gold Primary file can shape a double curved canal faster if a glide path is present, but it will take less time to reach working length if it is the only file used. Although it appears to be much safer to perform a glide path before using reciprocal shaping instruments, the statistical analysis shows that glide path had no real influence in the ability of WaveOne Gold Primary to reach working length. Further studies are needed to explore this idea and to see if reciprocal shaping instruments perform as well in dentin without a glide path.

## Figures and Tables

**Table 1:** Descriptive statistics for GP_time in PGG and WOGGG.

GP_Time (s)	Mean	SD	Min	Max	*p*-Value
PG	5.2005	1.38373	2.92	8.95	0.089
WOGG	4.5555	0.8872	3.02	6.63	

GP_Time, glide path preparation time; SD, standard deviation; PG, ProGlider; WOGG, WaveOne Gold Glider.

**Table 2:** Descriptive statistics for Shape_Time, Total_Time, RFWL and NPM in NGP, PG and WOGG groups.

Variable	NGP				PG				WOGG			
	Mean	SD	Min	Max	Mean	SD	Min	Max	Mean	SD	Min	Max
Shape_time (s)	17.1682	3.99178	12.58	29.29	14.8668	2.64826	11.08	21.67	13.1847	1.9147	9.04	15.85
Total_time (s)	17.1682	2.99178	12.58	29.29	20.0805	3.24897	15.31	28.73	17.2984	2.52387	12.65	22.37
RFWL	0.85	0.36635	0	1	0.95	0.22361	0	1	0.95	0.22361	0	1
NPM	9.7059	1.15999	8	12	8.2632	1.36797	6	12	7.0526	1.02598	4	8

NGP, no glide path; PG, ProGlider; WOGG, WaveOne Gold Glider; SD, standard deviation; RFWL, reach full working length; NPM, number of pecking motions.

**Table 3:** Turkey’s Test results for multiple comparisons for Shape_Time, Total_Time and NPM.

		Shape_Time (s)		Total_Time (s)		NPM	
Group		Mean Difference	*p*-Value	Mean Difference	*p*-Value	Mean Difference	*p*-Value
NGP	PG	2.30139	0.058	−2.91229	0.028	1.44272	0.002
	WOGG	3.98350	0.000	−0.53019	0.879	2.65325	0.000
PG	NGP	−2.30139	0.058	2.91229	0.028	−1.44272	0.002
	WOGG	1.68211	0.190	2.38211	0.074	1.21053	0.008

NPM, number of pecking motions; NGP, no glide path; PG, ProGlider; WOGG, WaveOne Gold Glider.

## Data Availability

Dataset available on request from the authors.
